# Evaluation of a digital Dementia Game to promote public awareness about dementia in Singapore

**DOI:** 10.1186/s12889-025-22988-7

**Published:** 2025-05-21

**Authors:** Peter Kay Chai Tay, Tara Anderson, Jazlyn Seng Jia Ying, Elaine Kee Chen Siow, Stephanie Craig, Gary Mitchell, Christine Brown Wilson

**Affiliations:** 1 Singapore Institute of Technology, Health and Social Sciences, Singapore, Singapore; 2 School of Nursing and Midwifery, Queen’s University Belfast, Belfast, UK

**Keywords:** Dementia, Health, Education, Public health, Serious game, Gamification

## Abstract

**Background:**

The study aimed to evaluate the effectiveness of a “Dementia Game” in increasing awareness and improving attitudes towards dementia among the general public in Singapore. The game requires players to navigate a path and respond to questions related to misconceptions about dementia.

**Methods:**

Using a quasi-experimental pre-test/post-test design, 138 participants completed questionnaires before and after playing the digital game to assess changes in their attitudes towards dementia. The Attitudes towards Dementia Questionnaire (ADQ) was used to measure overall attitudes as well as the “hope” and “person-centred” subscales. Wilcoxon signed-rank tests were conducted to determine if there were statistically significant changes in ADQ scores from pre-test to post-test. Subgroup analyses were also performed to investigate differences based on participants’ prior experience with dementia.

**Results:**

Participants showed statistically significant improvements in their overall attitudes towards dementia, as well as in the “hope” and “person-centred” subscales, after playing the game. However, those who had prior experience working with people with dementia or had received dementia training did not show significant improvements.

**Discussion:**

The study demonstrates the potential of a digital game to promote public awareness and improve attitudes towards dementia. The interactive and educational features of the game were well-received by participants, suggesting it can be an effective tool for challenging stereotypes and stigma surrounding dementia. However, the lack of significant improvements among those with prior dementia experience or training may reflect a ceiling effect, as they likely had higher baseline knowledge and less room for improvement. Future research should explore the long-term impacts of the game and compare its effectiveness to other interventions, potentially using a randomised controlled trial design.

## Background

Dementia is an umbrella term which encompasses a range of progressive neurological illnesses causing cognitive and behavioural symptoms [1]. Although progression will vary between individuals, common symptoms include memory loss, communication and reasoning problems, changes in personality, and a reduction in ability to carry out daily activities [2]. The impact of dementia represents a significant global healthcare priority with more than 55 million individuals living with dementia worldwide, and nearly 10 million new cases emerging annually [3].

Despite this high prevalence and the substantial impact of the condition on those living with dementia, their family, friends, and healthcare providers, public understanding of the condition remains low [4]. For example, two in three of 70,000 respondents from 155 countries to an Alzheimer’s Disease International (ADI) Global survey on attitudes to dementia believed dementia to be caused by normal ageing [4]. This lack of public awareness hinders the ability of those living with Dementia to live active and fulfilling lives, with many experiencing prejudice and discrimination from both the public and healthcare professionals [5, 6].

In Singapore, dementia was initially referred to as 痴呆症 (chī dài zhèng) in Chinese, which carries negative connotations associated with insanity and idiocy. This term has been replaced by 失智症 (shī zhì zhèng), meaning “cognitive impairment disorder,” which has a less negative connotations. One in ten individuals aged 60 and above are living with dementia in Singapore [7]. A Singapore-wide survey found that both those living with dementia and their carers experienced rejection, loneliness and shame, with more than 30% of caregivers reporting feelings of embarrassment when providing care for their loved ones in public [8]. Another Singaporean study found a deficiency in knowledge about dementia correlated with misguided attitudes [9]. Due to the ageing population and growing number of people living with dementia, it is imperative to educate the public about the condition to improve knowledge and support for people living with dementia.

Initiatives to help address the negative stigma experienced by those living with dementia include Dementia Friendly Communities [10], “Forget Us Not” [11], and the Dementia Friends programme [12]. However, there remains a need for continual improvement of public awareness to reduce discriminatory behaviour towards those with dementia. Gamification represents a novel and innovative method of promoting public awareness and may be more effective in terms of learning and knowledge retention than conventional instruction [13]. ‘Serious games’ are games which serve a specific educational purpose within an entertaining game format. Such games have become increasingly used within healthcare education [14] and have been found to, for example, improve awareness of pancreatic cancer symptoms [15], and influenza vaccination uptake [16].

A serious game aimed at improving the general public’s attitudes towards dementia was previously co-designed with people with dementia [17]. This ‘Dementia Game’ was found to elicit statistically significant improvements in attitudes toward dementia with participants indicating more optimistic perceptions of the abilities and future potential of people with dementia following evaluation with over 1,000 participants from Northern Ireland [17]. Since this evaluation, the ‘Dementia Game’ has been used by over 6,000 players across 57 countries. Whilst 50% of these players come from the UK, 12% come for European countries, 7% come from the US with 25% of players coming from countries across the SE Asia region with the largest percentage of players in Indonesia (8.84%), Singapore (7.52%) and Malaysia (5.79%). However, further research is required to investigate the game’s effectiveness in different cultures. Therefore, the present study aims to evaluate the impact of the ‘Dementia Awareness Game’ on attitudes towards dementia within the general public in Singapore. The objectives of this study were:

1. To determine if playing a short Dementia Awareness Game influences the public’s attitudes towards dementia in Singapore
2. To evaluate the acceptability of the ‘Dementia Awareness Game’ as an educational tool to promote dementia awareness to the general public in Singapore.

## Methods

### Design/setting/population

A quasi-experimental pre-test/post-test design was used to compare public attitudes towards dementia before and after playing the ‘Dementia Awareness Game’. Questionnaires were delivered immediately prior to and following participation in the game. The study was conducted using convenience sampling of members of the public with recruitment via social media as well as promotion of the study by Dementia Singapore and emails to staff and students at the Singapore Institute of Technology.

### Intervention

The ‘Dementia Game’ was co-designed with people living with dementia who highlighted societal misperceptions about the activities and capabilities of people living with dementia and felt that with great awareness, the public could support people living with dementia to enjoy greater independence within the community [18]. Through the codesign process, a question bank was developed covering three themes: common symptoms of dementia; misconceptions about dementia; and abilities of people living with dementia to confront and dispel stereotypes and stigma surrounding dementia [17]. The game play is simple requiring a figure to navigate a path with obstacles in a set amount of time. Questions appear in random order and the clock stops once the question appears. After the player has submitted their response, the correct answer with an explanation is given to enhance the player’s knowledge. As questions are answered correctly, the player gains points to advance along the path until they reach the finish line. The Game can be completed in approximately three minutes. The player is allowed multiple attempts to improve their score, which places them on a leaderboard. The game is a HTML5 web-based application which can be played on any device with an internet connection and can be freely accessed here: www.dementiagame.com.

### Survey instrument

The pre- and post-measures were completed on Qualtrics. The pre-questionnaire recorded demographic details (sex, age, ethnic group, whether they had a family member or close friend living with dementia, whether they worked with people living with dementia, and whether they had previously undertaken dementia training). This was followed by the 19-item Approaches to Dementia Questionnaire (ADQ) [19, 20]. The ADQ has been validated with Singaporean health workers with a Cronbach’s alpha of 0.80 and intra-class coefficients of 0.65 [21].

This questionnaire consists of two domains related to ‘hope’ and ‘person-centred’ approaches. The ‘hope’ domain demonstrates either an optimistic or pessimistic approach to a person with dementia (e.g. people with dementia are sick and need to be looked after). The ‘person-centred’ domain focuses on how a person with dementia is seen as an individual person and their capabilities (e.g. it is important to respond to people with dementia with empathy and understanding). Each item of this questionnaire consisted of a five-point Likert scale ranging from strongly agree (1) to strongly disagree (5).

The post-questionnaire repeated the ADQ and included an evaluation of the game. Participants were asked to rate their overall satisfaction with the game on a scale from very dissatisfied (1) to very satisfied (5). Participants were also asked the extent to which the game effectively raised their awareness and understanding of dementia by selecting one of three responses: No, yes to some extent, or yes to a great extent. The user-friendliness of the game was then assessed via participants responses of ‘yes, very straightforward and clear’, ‘yes, with some difficulty’, or ‘no, they were confusing’. Participants also rated their likelihood of recommending the game to others from very unlikely (1) to very likely (5). Finally, participants were asked to provide feedback by answering open-ended questions about the game. These sought insights into aspects of the game they found enjoyable, informative, and suggestions for improvement.

### Data collection

Data collection for this study took place over a five-month period from July to October 2023. Interested individuals accessed the study through a Qualtrics link or QR code provided by the research team. As this study was open to the general public in Singapore, there were no inclusion/exclusion criteria. Within the Qualtrics platform, a participant information sheet was embedded outlining the details of the study. Participants were required to click a box to confirm they had read the information and consented to participate in the study. If participants did not consent to participate, they could still play the game without participating in the research study. All participants were permitted to withdraw from the study at any stage without giving any reason and information about these processes was provided within the participant information sheet. The game, and the pre- and post-test measures were embedded within Qualtrics, and the questionnaires were completed immediately prior to and following engagement with the game. Participants played the game individually on a device of choice at a time and location of their choosing. After playing the game, participants were directed to the post questionnaire. 

### Ethics

Singapore Institute of Technology Institutional Review Board granted ethical approval for this study (Ref: 2023063) after considering benefits and risks and ensuring participants autonomy would be respected. All methods were performed in accordance with the Declaration of Helsinki [22].

### Analysis

The pre- and post-test datasets were matched prior to analysis using email addresses which served as an identifier for each participant between the two datasets. All analyses were conducted in SPSS version 29. Participants who did not complete the post-test questionnaire were removed from the final dataset.

Total scores for each questionnaire sub-scale were calculated based on Likert scale responses for both the pre- and post-test questionnaires. Several items were reverse coded to ensure higher scores reflected more positive attitudes toward dementia. Descriptive statistics were performed to observe demographic details of the participant sample. As the data did not meet the assumption of normality required for a paired-samples t-test, non-parametric Wilcoxon signed-rank tests were conducted to examine the change from pre-test to post-test for both domains of the ADQ as well as the total ADQ. Descriptive statistics were also used to examine differences based on whether participants had a family member or close friend living with dementia, whether they worked with people living with dementia, and whether they had undergone previous dementia training. Finally, responses to the game evaluation questions were analysed descriptively.

The qualitative data obtained from responses to the open-ended questions presented on the post-test questionnaire were analysed using Braun and Clarke’s six step thematic analysis [23]. One researcher (J) familiarised themselves with the data and generated individual codes and combined these codes into themes which were reviewed with a second researcher for rigor (P).

## Results

In total, 321 participants were recruited to evaluate the impact of the ‘Dementia Game’ on attitudes toward dementia as assessed by pre- and post-questionnaires. However, 183 participants were excluded for various reasons including failure to provide informed consent, failure to engage with the game, and missing pre- or post-test data. The final dataset, therefore, comprised 138 participants. The subsequent analysis and findings are based on this refined participant group.

### Demographic details

Table 1 provides participant demographics of those included in paired t-test analysis (*N* = 138). Most participants were female (61.6%), Chinese (89.1%), and aged 24 or younger (35.5%).

**Table 1:** Participant demographics

		**N**	**%**
Gender	Female	85	61.6%
	Male	51	37.0%
	Prefer Not to Say	2	1.4%
Age (years)	18–24	49	35.5%
	25–34	44	31.9%
	35–44	24	17.4%
	45–54	16	11.6%
	55–64	5	3.6%
Ethnic Group	Chinese	123	89.1%
	Malay	5	3.6%
	Indian	7	5.1%
	Other	3	2.2%

The participants were also asked to indicate any previous knowledge and/or experience of dementia. Most of them had neither a family member or close friend living with dementia, nor had worked with people living with dementia. Only 8% of the participants had undertaken dementia training. Further descriptive statistics regarding participants’ experience is presented in Table 2.

**Table 2:** Participant knowledge and/or experience of dementia descriptive statistics

	**N**	**%**
I have a family member or close friend living with dementia	53	38.4%
I work with people living with dementia	23	16.7%
I have previously undertaken dementia training	11	8%

### Pre-test to post-test changes in ADQ scores

Participants showed increased scores on the overall ADQ at post-test (*M* = 72.28, *SD* = 8.42) compared to their pre-test score (*M* = 67.56, *SD* = 6.98), as shown in Table 3. A Wilcoxon signed-rank test was used to determine whether there was a statistically significant mean difference between pre- and post-test overall ADQ scores, this found a statistically significant mean increase*, z* = −6.40, *p* <.001. Statistically significant mean increases were also found for both the hope (*z* = −7.09, *p* <.001) and person-centred (*z* = −5.41, *p* <.001) subscales, as detailed in Table 3.

**Table 3:** Wilcoxon signed-rank test of pre- and post-test ADQ scores

	**Pre-test Mean (*SD*)**	**Post-test Mean (*SD*)**	***Z***	**Sig. (2-tailed)**
Total ADQ score	67.56 (6.98)	72.28 (8.42)	−6.40	<.001
Hope subscale	23.34 (4.01)	25.99 (4.89)	−7.09	<.001
Person-centred subscale	44.22 (4.39)	46.49 (5.22)	−5.41	<.001

### Group differences based on previous experience

Further Wilcoxon signed-rank tests were conducted to investigate any differences in subgroup score increases based on previous experience. Statistically significant increases in total ADQ scores were observed irrespective of whether participants had a family member or close friend living with dementia. Table 4 presents details of between-group differences on baseline ADQ scores between those with and without previous knowledge and/or experience of dementia.

**Table 4:** Wilcoxon signed-rank test subgroup analysis

		Total score (SD)		*z*	Hope subscale (SD)		*z*	Person-centred subscale (SD)		*z*
		Pre	Post		Pre	Post		Pre	Post	
I have a family member or close friend living with dementia	No	67.36 (6.38)	72.47 (8.18)	−5.70	23.45 (3.92)	26.24 (4.74)	−5.47	43.92 (4.08)	46.24 (5.16)	−4.70
	Yes	67.87 (7.92)	71.98 (8.86)	−2.97	23.17 (4.19)	25.60 (5.15)	−4.38	44.70 (4.85)	46.38 (5.37)	−2.63
I work with people living with dementia	No	67.43 (6.72)	72.21 (8.15)	−6.77	23.22 (3.94)	26.02 (4.85)	−7.16	44.22 (4.28)	46.19 (5.04)	−5.17
	Yes	68.17 (8.33)	72.65 (9.85)		23.96 (4.41)	25.87 (5.20)		44.22 (5.04)	46.78 (6.13)	
I have previously undertaken dementia training	No	67.06 (6.80)	72.11 (8.35)	−6.73	23.13 (3.92)	25.96 (4.91)	−7.40	43.93 (4.32)	46.15 (5.17)	−5.34
	Yes	73.27 (6.87)	74.27 (9.34)		25.73 (4.50)	26.36 (4.86)		47.55 (3.98)	47.91 (5.82)	

Participants who worked with people with dementia displayed higher mean baseline ADQ total scores (*M* = 68.17, *SD* = 8.33), compared to those who did not work with people with dementia (*M* = 67.43, *SD* = 6.72). Those who worked with people with dementia did not show statistically significant improvements in ADQ scores (*p* = 0.824) in contrast to those without such experience (*z* = −6.77, *p* <.001).

Similarly, individuals who had previously undertaken dementia training exhibited higher baseline ADQ total scores (*M* = 73.27, *SD* = 6.87) compared to those without such training (*M* = 67.06, *SD* = 6.89). Those with prior training did not demonstrate statistically significant score improvements (*p* = 1.00), in comparison to those without prior dementia training (*z* = −6.73, *p* <.001).

### Game evaluation

Table 5 presents a summary of game evaluation data. Most participants expressed satisfaction with the game (59.9%), while a significant portion felt neutral (31.4%), and a small percentage showed dissatisfaction (8.7%) The game was perceived as effective in enhancing awareness and understanding of dementia by most participants (95.6%), of which 25.5% felt that their understanding greatly improved. Additionally, the majority found the game easy to comprehend and navigate (62.5%) and would be likely to recommend the game to others (53.7%).

**Table 5:** Game evaluation descriptive statistics

**Item**	***N***	**%**
How would you rate your overall satisfaction with the game?		
Very dissatisfied	1	0.7
Dissatisfied	11	8.0
Neutral	43	31.4
Satisfied	76	55.5
Very satisfied	6	4.4
Did the game effectively raise your awareness and understanding of dementia?		
Yes, to a great extent	35	25.5
Yes, to some extent	96	70.1
No	6	4.4
Was the game easy to understand and follow?		
Yes, very clear and straightforward	85	62.5
Yes, with some difficulty	41	30.1
No, they were confusing	10	7.4
How likely are you to recommend this game to others?		
Very unlikely	5	3.7
Unlikely	13	9.6
Neutral	45	33.1
Likely	63	46.3
Very likely	10	7.4

### Qualitative feedback

Qualitative data was obtained in the form of positive feedback and suggestions for improvement. Four themes emerged in relation to positive feedback while five were found for suggestions for improvement, as shown in Table 6.

**Table 6:** Qualitative themes

**Positive Feedback**	**Suggestions for Improvement**
Interactive features	Content and information
Educational content	Gameplay and interface
User experience and design	Scenario and storyline
Awareness and perspective	Rewards
	Localisation and language

#### Positive feedback

##### Interactive features

Participants engaged positively with the interactive features, finding enjoyment in the diverse aspects of gameplay. The quiz-like format, coupled with multiple-choice questions, allowed for dynamic interactions. Participants expressed that they liked the “interactive questions”, “explanation pop-ups”, and “quiz-like style”. The incorporation of a points system and the autonomy to choose directions during gameplay contributed to an engaging and gamified learning experience. Participants appreciated this freedom, “I like that we were given choices about which direction to move in”, “liked the ability to choose my route”, and felt “the pathway is encouraging me to continue playing”.

##### Educational content

Participants appreciated the educational content, particularly the immediate feedback of facts and explanations provided after each question. Comments included: “It gave you the answers to the questions so you can learn from them” and “the clarification after each answer is informative”. The short and informative nuggets of information, presented in quiz format, facilitated a better understanding of dementia. Praise was given for delivering essential facts in an easily comprehensible manner, including succinct questions and answers, and gamified quiz elements for effective knowledge acquisition. Participants commented: “the use of questions in the form of common myths… helped to debunk them” and “the questions… helped you to reinforce your knowledge and understanding of dementia”.

##### User experience and design

The colourful and visually appealing interface was well-received. The variety of knowledge presented in a short and concise manner added to the positive user experience. Comments included: “questions and answers are short and concise”, “it was colourful, bite-sized info”, and “facts and misconceptions were well relayed”.

##### Awareness and perspective

The game played a significant role in raising awareness and providing different perspectives on dementia. Participants expressed a deeper understanding of the prevalence of dementia among people around them and emphasised the importance of understanding the needs of people with dementia. Participants commented: “the questions made me think more about dementia”, “the statistics and questions asked are thought provoking”.

#### Suggestions for improvement

##### Content and information

Participants suggested enhancing the educational value of the game by including information about symptoms of dementia. There was a call to make content less statistical and more relevant to the general public, tailoring it to Singapore and Asia-specific contexts, for example one participant commented: “It would be good to include information such as where people can get help in Singapore should the players suspect themselves or their loved ones are developing dementia symptoms”. Other recommendations included: adding “links or articles after each question if a person wants to know more about an aspect of dementia” and “incorporating content on how to interact with someone with dementia”.

##### Gameplay and interface

To improve the user experience, participants proposed implementing a forced tutorial at the start of the game, with the option to skip if desired. Suggestions included simplifying the path to advance, adjusting the countdown time as the current time limit may be “too stressful”, integrating more minigames, ensuring mobile-friendly interactions, and enhancing sound effects and music. For example, one participant suggested: “for each wrong answer to take a step back”. Varying opinions on the music were expressed as some felt it was “too distracting” and others “love[d] the calm music”.

##### Scenarios and storyline

Participants desired an enhanced gaming experience, suggesting it “would have been better is a scenario based interaction was involved” and “perhaps show various scenarios when interacting with a person with dementia”. The inclusion of a storyline involving people with dementia was suggested to offer a more immersive and emotionally engaging experience. Clearer objectives and an overarching end goal were proposed to provide players with a more purposeful experience. Additionally, participants called for the incorporation of profiles, videos, or pictures to personalise the game and create a deeper connection. Comments included: “Perhaps can add in an end goal, make a profile that makes it more personalised” and “not very sure what was the end goal”.

##### Rewards

Participants proposed refining the rewards system within the game. Suggestions included providing more rewards, introducing a point multiplier for correct answers, along with additional bonuses at certain milestones. Participants commented: “It would be good to include point multiplier effect”, and “points could be exchanged for perks/special skills… and encourage people to replay the game”. Some participants also proposed the inclusion of “penalties for getting questions wrong”, or the introduction of a multiplayer mode for added competition.

##### Localisation and language

Recognising the diverse audience, participants recommended making the game more suited to the Singapore context. Including multiple languages to broaden accessibility and cater to a more global audience was suggested to ensure that the gaming experience resonates with players from different linguistic backgrounds.

## Discussion

Increased public education of dementia may help to address the negative stigma and associated discrimination and prejudice experienced by people living with dementia [5, 6]. Therefore, the present study aimed to evaluate a serious game as an educational tool within the Singaporean context. While the ‘Dementia Game’ has undergone evaluation in prior research [17], this was the first study to explore its impact in Singapore. Following engagement with the game, participants showed improvements in participants’ overall attitudes towards dementia, as well as in the “hope” and “person-centred” subscales, after playing the game, highlighting the potential of this ‘Dementia Game’ as an educational tool. However, those with prior experience working with people with dementia or dementia training did not show significant improvements. Most participants expressed satisfaction with the game and found it effective in raising awareness and understanding of dementia.

In line with previous research in which the ‘Dementia Game’ elicited more positive attitudes toward dementia, participants in this study showed increased scores across both ‘hope’ and ‘person-centredness’ [17]. This reflects the game’s impact in promoting optimistic views of the abilities and capabilities of people with dementia as well as recognition of those with dementia as unique individuals with hopes and values. This may be explained by conceptual reframing as participants gained a more positive and nuanced understanding of the condition via the education they received through engagement with the game, an integral aspect of reducing stigma [5, 24]. Younger people in other Asian countries have been found to have a greater level of digital literacy having grown up with smart devices and the internet [25]. As the majority of participants were aged between 18 and 34, this may account for the interest in the digital game as a mechanism for raising dementia awareness, similar to previous studies [17].

Individuals who had either worked with people with dementia or undertaken dementia training previously, however, were not found to exhibit the same level of improvement in scores following engagement with the game. This contradicts previous findings that the game demonstrated significant improvements in attitudes irrespective of professional involvement with dementia or prior dementia training [17]. Therefore, findings may reflect the increased baseline knowledge of these groups, and a resulting ceiling effect. It is plausible that those with prior training and/or experience had less room for improvement due to their higher baseline knowledge. This is in line with previous research in which increased contact with people with dementia and prior training lead to more positive attitudes toward dementia including more person-centred attitudes [19, 26]. Similarly in a study assessing the attitudes of community healthcare workers in Singapore, using the ADQ, having a family member or experience in caring for people with dementia did not translate into a higher score [21]. Although the same study revealed generally positive attitudes towards people living with dementia with a mean ADQ score 68.4 out of 95 [21], which was similar to the pre-test scores in this study.

Participants highlighted various positive features of the ‘Dementia Game’, underscoring its efficacy in challenging stereotypes and stigma surrounding dementia. Participants commented on the integration of fun and interactive gamification elements within the educational context. The interactive gameplay. Involving gamified quiz elements, map navigation, and decision-making regarding directions and routes helped to actively engage participants. Further, the point system served as a mechanism for positive reinforcement and reward-based learning [27, 28].

The content of the ‘Dementia Game’ was determined by co-design with people with dementia. This allowed the priorities and personal experiences of those affected to be incorporated into the game’s design. Other co-designed games have also found similar effects of increased awareness, and positive evaluation by users [15, 29]

The content of the ‘Dementia Game’ was determined by co-design with people with dementia. This allowed the priorities and personal experiences of those affected to be incorporated into the game’s design, ensuring the game captured their journey, directly challenged stereotypes, and fostered a more profound understanding among players. Other co-designed games have found similar effects of increased awareness, and positive evaluation by users [15, 29]. Evaluating the dementia game in Singapore provided helpful feedback in what was needed for cultural adaptation. For example, players suggested different languages as Singapore is a pluralist society and more structured content to engage players in the experience of the dementia journey. Whilst this game was successful in challenging stereotypes and fostered a more profound understanding of dementia, creating scenarios with interactive games has also been identified as a mechanism to improve dementia awareness in other Asian countries and with UK children’s nursing students [30]. Future dementia awareness game development should also consider the perspectives of people living with dementia and their carers in Singapore.

### Strengths and limitations

The strengths for the present study include the adoption of a mixed methods approach which provided qualitative insights to complement the quantitative data collected. This provided a more holistic understanding of the game’s effectiveness in evoking changes in attitudes towards dementia.

This study is also subject to limitations including participant drop-out which resulted in low statistical power and limited the ability to detect small or moderate effects. This reduced sample size also limits the generalisability of findings to the broader population. The majority of participants identified as ethnic Chinese which further limits the generalisability of findings to more ethnically diverse settings as attitudes toward dementia can be significantly impacted by cultural factors that vary among different ethnic groups. In addition, as the game predominantly focused on introductory and basic-level information, participants with prior exposure to dementia training or working with people with dementia may not have found the content challenging or stimulating enough to evoke attitudinal changes. More personalised or advanced interventions may be required for this population.

In terms of future research, it would be useful to extend the current findings by examining the long-term impact of engaging with the ‘Dementia Game’. This could help to shed light on learning retention and any sustained impact the increased positive attitudes. In addition, it may be helpful to compare the impact of the serious game with other interventions. Although the pre- and post-test design of the present study represents a pragmatic and cost-effective method of evaluating the intervention, this design does not have a comparison or control group. Therefore, future research would benefit from testing the effectiveness of the ‘Dementia Game’ in a randomised control trial (RCT).

## Conclusion

It is evident that the ‘Dementia Game’ shows promise as an educational tool to improve attitudes towards dementia, particularly among individuals who lack prior training and/or experience in the field. Additionally, participant feedback highlights the positive experience of those who engaged with the game and provides suggestions for improvements which may be considered to enhance the game. Future research may benefit from increased sample size of a more ethnically diverse population and exploration of the long-term impacts of the game.

## Data Availability

The datasets used and/or analysed during the current study are available from the corresponding author on reasonable request.
